# Supplementary material for: Complexome profiling of the Chlamydomonas psb28 mutant reveals TEF5 as an early PSII assembly factor
Source: Plant Cell. 2025 Mar 17;37(6):koaf055. doi: 10.1093/plcell/koaf055 (PMC12164590; doi:10.1093/plcell/koaf055)
Supplement: koaf055_Supplementary_Data [file koaf055_supplementary_data.zip › psb28_interactive.zip!/plots/intensity/qualityPlots/peptides/PLAP10_Cre03.g189300.t1.1.html]

### Cre03.g189300.t1.1

Trivial name: PLAP10  
SeqCoverage [median]: 9.60  
Mass: 32.99 kDa  
  
 

| Sequence | MissedCleavage | Mass [Da] | wtIntensity | muIntensity | Score |
| --- | --- | --- | --- | --- | --- |
| DAVKPLQDATK | 1 | 1184.64 | 0.00 | 668.75 | 72.34 |
| GDGGAVYIFK | 0 | 1025.52 | 0.00 | 782.94 | 54.16 |
| GDGGAVYIFKK | 1 | 1153.61 | 0.00 | 1090.13 | 65.50 |
| RLQFTVER | 1 | 1047.58 | 0.00 | 321.96 | 40.00 |
|  |  |  | 0.0 | 2863.8 |  |
